# Education Research: Assessing the Current State of Telestroke Exposure in Vascular Neurology Fellowship

**DOI:** 10.1212/NE9.0000000000200366

**Published:** 2026-09-15

**Authors:** Nikita Chhabra, David S. Liebeskind, Amy Katherine Guzik, Brian Stamm, Erika Layne Weil, Darin B. Zahuranec, Mollie McDermott, Robert Miller, Devin L. Brown

**Affiliations:** 1 Department of Neurology, University of Michigan, Ann Arbor, MI;; 2 Department of Neurology, University of Southern California, Los Angeles, CA;; 3 Department of Neurology, Wake Forest University School of Medicine, Winston-Salem, NC; and; 4 VA Center for Clinical Management Research and Geriatric Research Education and Clinical Center, VA Ann Arbor Healthcare System, Ann Arbor, MI.

## Abstract

**Background and Objectives:**

Telestroke is now a core component of acute stroke care, yet how fellows are trained to deliver care in this setting remains variable. With telestroke incorporated into Accreditation Council for Graduate Medical Education (ACGME) milestones, there is a need to better understand current training practices and gaps. The objective of this study was to evaluate telestroke training across vascular neurology (VN) fellowship programs in the United States and identify barriers to fellow participation.

**Methods:**

We conducted a national survey of VN fellowship program directors identified through the Fellowship and Residency Electronic Interactive Database Access (FREIDA) database. The survey assessed program characteristics, fellow involvement in telestroke, educational modalities, and perceived barriers. Responses were analyzed using descriptive statistics.

**Results:**

Of 115 program directors contacted, 67 (58.3%) responded. Most programs (92.5%) reported offering telestroke services, and the majority included fellows in clinical encounters, primarily through video (73.3%) and telephone (56.7%) consultations. However, structured educational components were less common, with 48.3% offering telestroke-specific skills training, 25.0% offering didactics, and 11.7% incorporating simulations. Although 71.7% of program directors felt fellows were adequately prepared for independent practice, 28.3% reported uncertainty or lack of readiness. Common barriers to telestroke fellow involvement included supervision requirements, credentialing challenges, and limited administrative support, with nearly two-thirds of programs identifying unmet resource needs.

**Discussion:**

Telestroke training in VN fellowships is common but varies in structure, including the availability of formal didactics, simulation, skills-based training, supervision models, and barriers to fellow participation. Defining competency-based expectations, while allowing for flexible and scalable training models, may help address variability and better prepare fellows for independent telestroke practice.

## Introduction

Telestroke has become an essential component of modern stroke care, enabling neurologists to provide safe and effective acute stroke treatment in a remote manner.^1,2^ Telestroke can promote equitable access to timely care,^3^ particularly for patients in underserved or remote areas.^4,5^ It is imperative that vascular neurology (VN) fellows are adequately trained to meet the growing demand for specialists proficient in providing remote stroke care. These factors underscore the need for formal and standardized telestroke training to be integrated into VN fellowship programs. A recent review supports this assertion, emphasizes the importance of structured telestroke education, outlines key components of such training, and proposes metrics for evaluating fellow competency.^6^

The 2021 ACGME Vascular Neurology Milestones introduced a specific telestroke milestone, with a level 4 expectation (the target level for graduation, indicating readiness for independent practice) that fellows' conduct stroke consultations using remote technology^7^ (full milestone available in eTable 1). However, there remains limited curricular guidance on if or how fellows are expected to achieve this milestone before graduation. Specifically, it remains unclear how VN fellowship programs operationalize telestroke training in practice, including the extent to which fellows participate in telestroke encounters, the educational modalities used to prepare them, the supervision models applied during clinical encounters, and the institutional barriers that limit fellow integration.

### Objective

The objective of this study was to characterize the current landscape of telestroke training in VN fellowship programs in the United States, including fellow participation, educational modalities, supervision models, perceived preparedness for independent practice, and barriers to broader implementation.

## Methods

### Definitions

For the purposes of this study, telestroke is defined as the use of synchronous 2-way audio or video conferencing to deliver virtual acute stroke care with patients and/or consulting providers.

### Setting and Participants

Current VN fellowship directors were identified through FREIDA (American Medical Association Residency and Fellowship Database).^8^ VN program directors were offered participation through an initial email invitation followed by 2 reminder emails.

### Survey Development

An online survey was developed and distributed using the Qualtrics platform. The survey was developed through an iterative process involving a VN fellow and VN faculty including fellowship program directors, telestroke program directors, and additional VN faculty with independent telestroke practice and fellow supervision experience across 3 academic institutions. The survey was not formally pilot tested.

The survey questions were organized by section to include program characteristics, current state of fellow telestroke exposure, and perceived barriers to expanding fellow integration. Program characteristics included geographic location, number of VN fellows and faculty, institutional stroke center designation, and the scope and duration of telestroke services, such as consultation modalities and the number of spoke hospitals covered. The section on fellow telestroke exposure explored how fellows engage with telestroke (e.g., simulation, didactics, and direct patient assessments through video or telephone), the supervision models used during these encounters, and whether dedicated telestroke training is provided at the start of fellowship. Respondents were also asked if their programs offer formal lectures on telestroke-related topics and whether they believe current training adequately prepares fellows for independent practice. Finally, the survey assessed perceived barriers to expanding telestroke involvement of VN fellows, including credentialing challenges, faculty limitations, and technologic costs, as well as resources or supports that might facilitate greater fellow participation. All responses were collected anonymously. The complete survey instrument is included in eAppendix 1 for reference.

### Data Analysis

Descriptive statistics were used to summarize survey responses.

### Standard Protocol, Approvals, Registrations, and Patient Consents

This study was deemed exempt under Exemption 2 (1) and/or 2 (2) of 45 Code of Federal Regulations 46.104(d) by the University of Michigan Institutional Review Board.

### Data Availability

Anonymized data not published within this article will be made available by request from any qualified investigator.

## Results

### Overall Results and General Program Characteristics

Of the 115 VN fellowship program directors invited to participate, 67 (58.3%) responded. Program characteristics are summarized in Table 1. Responding programs were geographically diverse (eTable 2), with most offering 2 or 3 ACGME-accredited fellowship positions per year and having 10 or fewer VN faculty. Nearly all programs (98.5%) reported designation as a Comprehensive Stroke Center.

**Table:** Vascular Neurology Fellowship Program Characteristics (67 of 115 Respondents, 58%)

	n (%)	% missing
Geographic location		
Midwest	19 (28.8)	1.5
Northeast	18 (27.3)	
South	17 (25.8)	
West	12 (18.2)	
Number of vascular neurology faculty		
0–5	22 (33.3)	1.5
6–10	33 (50.0)	
≥11	11 (16.7)	
Stroke center designation		
JC Comprehensive Stroke Center	64 (97.0)	1.5
DNV Comprehensive Stroke Center	1 (1.5)	
No designation	1 (1.5)	
Number of ACGME-approved fellowship positions (%)		
1	10 (15.2)	
2	33 (50.0)	
3	11 (16.7)	
4	9 (13.6)	
5	3 (4.5)	
	Median (IQR)	2 (2–3)
Years providing telestroke services		
<1 y	1 (1.7)	10.4
1–5 y	8 (13.3)	
6–10 y	14 (23.3)	
≥11 y	35 (58.3)	
Don't know	2 (3.3)	
Number of spoke hospitals covered		
0–5	14 (23.3)	10.4
6–10	21 (35.0)	
11–20	12 (20.0)	
≥21	13 (21.7)	

Abbreviations: DNV = Det Norske Veritas; JC = Joint Commission.

### Telestroke Program Characteristics and Fellow Participation

Among responding programs, 62 of 67 (92.5%) reported that their institution provided telestroke services. Of these, 49.3% offered both video and telephone consultations, 28.4% offered video-only consultations, and 13.4% offered telephone-only consultations. More than half of programs providing telestroke services had performed so for 11 or more years (N = 35, 58.3%).

Programs reported a wide range in telestroke network size, with 35% covering 6–10 spoke hospitals, while 23.3% covered 0–5, 20.0% covered 11%–20%, and 21.7% covered 21 or more spoke hospitals.

Among programs offering telestroke services, most reported some level of fellow participation (55/60, 91.6%). The majority indicated that fellows participate in video-based telestroke consultations (73.3%) and telephone consultations with outside providers (56.7%). Nearly half of programs reported providing structured training in telestroke-specific skills at the start of fellowship (48.3%), whereas fewer offered formal didactics on telestroke-related topics (25.0%) or simulation-based training (11.7%).

Programs reported using multiple supervision models. The most commonly reported model involved fellows leading telestroke encounters with direct attending observation (81.8%). Many programs reported fellows shadowing attending-led encounters (61.4%) or shared fellow-attending interaction with the patient (59.1%). Nearly half of programs reported fellows leading telestroke encounters without direct attending observation (43.2%).

Most of the programs (71.7%) felt VN fellowship training adequately prepared fellows for independent telestroke practice, while 10.0% disagreed and 18.3% were unsure (eTable 3A).

### Perceived Barriers and Resources Needs for Expanding Fellow Telestroke Exposure

Most programs reported challenges affecting fellow participation in telestroke (67.8%). The most common challenges reported included need for direct supervision (27.1%), limited staff to manage credentialing (23.7%), and the need to establish program letters of agreement with spoke hospitals (20.3%). Additional reported barriers included the high cost of credentialing (16.9%), insufficient faculty involvement in telestroke (11.9%), lack of a telestroke network within the hospital system (8.5%), and the cost of HIPAA-compliant equipment for fellows (3.4%) (eTable 3, B and C).

Among programs identifying unmet needs (63.8%), the most commonly cited resource needs included additional funding for technology and infrastructure (31.0%) and dedicated staff to support the credentialing process (29.3%). Less frequently reported needs included enhanced faculty training and expertise in telestroke (13.8%), establishment of a telestroke network within the hospital system (10.3%), and partnerships with academic institutions with more established telestroke networks (8.6%).

## Discussion

In this national survey of VN fellowship program directors, telestroke exposure among fellows was common but heterogeneous. Although this variability may partially reflect differences in institutional infrastructure and clinical resources, formalized educational experiences were less consistently implemented. Only 48.3% of programs reported structured training in telestroke-specific skills, with even fewer offering didactics (25.0%) or simulation-based training (11.7%). These findings suggest that efforts should focus on defining core, competency-aligned educational expectations and promoting structured training approaches aligned with ACGME milestones.

Prior single-institution experiences provide insight into how structured telestroke education may address this variability. Jagolino et al.^9^ described a telestroke curriculum which includes timely completion of credentialing before fellowship start, structured telestroke-specific training, a dedicated telestroke rotation, defined supervision frameworks, and a telestroke-specific case conference. In a survey of their prior fellows, graduates strongly agreed that telestroke exposure during fellowship led to proficiency in performing telemedicine consultations. This finding is supported by existing literature demonstrating improvement in telestroke thrombolysis metrics with increasing consultation volume among both fellows and attending physicians, underscoring the role of ongoing experiential learning across stages of expertise.^10^ Furthermore, more than 80% of fellows surveyed endorsed having telemedicine as a required component of VN fellowship training.^10^ Collectively, these findings support the growing consensus around the educational value of telestroke.^6^

Despite a growing body of evidence for formalized telestroke training, our survey findings highlight substantial variability in telestroke training across VN fellowship programs. Although most programs reported fellow participation in patient-facing telestroke encounters, such as telephone-based (56.7%) and video-based (73.3%) consultations, far fewer reported structured exposure to non–patient-facing educational components, including telestroke-specific skills training (48.3%), formal didactics (25.0%), or simulation-based learning (11.7%). This pattern suggests that many fellows gain experiential exposure to telestroke without foundational preparation in the distinct technical, cognitive, and communication skills required for effective independent telestroke practice. Unlike in-person care, many of these skills are difficult to observe and deliberately practice during real-time clinical encounters, particularly in high-stakes, time-sensitive settings. These include conducting a focused neurologic examination without bedside access, directing on-site personnel, adapting empathetic communication to a virtual setting, and remotely obtaining consent for high-stakes treatments. Additional complexity arises from variability in spoke site resources and the need to coordinate transfers to higher levels of care. As a result, these competencies may not be reliably acquired through experiential learning alone. Structured non–patient-facing educational modalities (such as simulation, skills-based training, and targeted didactics) therefore provide a critical opportunity for deliberate practice in a lower-risk environment and may better prepare fellows for independent telestroke responsibilities.

A majority of programs reported both challenges affecting fellow participation and identified resources that may help expand fellow-level telestroke exposure. The most frequently cited obstacles, including the need for direct supervision (27.1%), limited staffing to support the credentialing process (23.7%), and the requirement to establish program-specific letters of agreement with spoke hospitals (20.3%) highlight the logistical complexity of integrating fellows into telestroke workflows. Notably, these requirements may vary across states and health systems, suggesting that some barriers may reflect local policy or institutional interpretation rather than universal standards. Additional barriers, such as the cost of credentialing, limited faculty involvement, and lack of an established telestroke network, further suggest that fellow participation is often constrained by institutional infrastructure rather than educational intent. Consistent with these challenges, nearly two-thirds of programs identified unmet resource needs, most commonly additional funding for technology and infrastructure (31.0%) and dedicated staff to manage credentialing (29.3%).

Together, these findings suggest that variability in telestroke exposure across fellowship programs likely reflects both differences in educational structure and significant administrative and infrastructural barriers. Although formalizing telestroke training through a standardized, competency-aligned framework may help define core educational expectations, achieving uniform implementation across all programs may not be feasible given resource variability. As such, alternative and scalable approaches may be needed, including interprogram collaborations, shared telestroke networks, partnerships with external telehealth services, and supplemental training modalities such as simulation-based curricula or virtual rotations.^6^ It is important to acknowledge that although these models may provide more equitable access to high-quality telestroke training while allowing programs to adapt to local constraints, they are also resource-intensive and may not be feasible for all programs.

Consistent with the variability noted, 24.4% of program directors reported lack of readiness or uncertainty regarding fellows' preparedness for independent telestroke practice at the end of training, underscoring the need for a structured, competency-based approach that clearly defines and assesses expected benchmarks. Telestroke training is embedded within ACGME milestones and should align with the 6 core competencies. Telestroke offers a unique setting in which all 6 competencies can be developed and assessed, but this requires deliberate curricular design; without it, training may remain experiential rather than competency-driven.^6^ Ultimately, preparing fellows for independent telestroke practice will require both clearly defined competency expectations and flexible, program-specific strategies to achieve them.

Data were self-reported by program directors and are therefore subject to response and recall bias. Programs with established telestroke programs may have been more likely to respond. This may have led to overestimation of the prevalence of fellow telestroke exposure, structured educational components, and perceived preparedness for independent practice while underestimating barriers among programs with less developed telestroke infrastructure. Although the survey language was developed iteratively by multiple vascular neurologists, the survey instrument was not formally pilot tested, which may have affected question interpretation or response consistency. In addition, the survey did not capture objective measures of fellow competency or case volume. Our survey also did not ask program directors to report the percentage of trainees achieving each level of the ACGME telestroke milestone. As a result, our findings describe the availability and structure of telestroke training but cannot determine the extent to which trainees achieve telestroke-related milestones or develop competency in this area. Despite these limitations, the favorable response rate and geographic diversity of programs provide a meaningful snapshot of current national practices.

In conclusion, telestroke training in VN fellowships is common but varies in structure, including the availability of formal didactics, simulation, skills-based training, supervision models, and barriers to fellow participation. These findings highlight the need to better define core components of telestroke training while recognizing that uniform implementation may not be feasible across programs with differing institutional resources. Flexible and scalable approaches, such as simulation-based curricula, interprogram collaborations, shared telestroke networks, virtual rotations, and partnerships with established telestroke programs, may help expand access to structured telestroke education. Future studies should evaluate competency-specific content, milestone attainment, and trainee outcomes to determine how best to prepare fellows for independent telestroke practice.

## Glossary

ACGME: Accreditation Council for Graduate Medical Education
VN: vascular neurology
